# Persistent Hyperhidrosis in the Psychiatric Setting: A Case Report

**DOI:** 10.7759/cureus.114349

**Published:** 2026-08-11

**Authors:** Ryan Ha, Syed F Qadri

**Affiliations:** 1 Psychiatry, Creighton University School of Medicine, Omaha, USA

**Keywords:** adrenergic modulators, secondary hyperhidrosis, serotonin-norepinephrine reuptake inhibitor (snri), temperature dysregulation by antipsychotics, tricyclic anti-depressants

## Abstract

Hyperhidrosis, defined as excessive sweating beyond what is required for thermoregulation, is a recognized adverse effect of numerous medications, including several classes of psychotropic agents acting through different mechanisms. We present the case of a 43-year-old male with a complex psychiatric and medication history who developed persistent, drenching night sweats that continued despite multiple psychotropic medication adjustments and discontinuations. Alternative metabolic, infectious, endocrine, and substance-related causes were considered without identification of a clear etiology. This case highlights the difficulty of attributing hyperhidrosis to a single agent in patients with complex medication histories and emphasizes the importance of balancing symptom management with the continuation of necessary psychiatric treatment.

## Introduction

Hyperhidrosis is a disorder characterized by eccrine gland overactivity beyond normal thermoregulation, which can occur as a primary condition or secondary to medications [1,2]. Psychotropic-induced hyperhidrosis is an often-underreported adverse effect observed across multiple medication classes, which include antidepressants, antipsychotics, and other centrally acting agents. Estimates suggest that 5-20% of patients receiving psychotropic medications experience clinically significant hyperhidrosis, although true prevalence may be underestimated due to inconsistent reporting [3]. While selective serotonin reuptake inhibitors (SSRIs) are frequently implicated, evidence indicates that serotonin-norepinephrine reuptake inhibitors (SNRIs), such as venlafaxine [4], carry a comparable risk, supporting a broader class effect among second-generation antidepressants. Antipsychotic medications such as clozapine, olanzapine, and quetiapine have also been associated with hyperhidrosis, with proposed mechanisms involving complex interactions at muscarinic and autonomic pathways regulating sweat production [5].

Persistent hyperhidrosis in psychiatric patients often represents a diagnostic challenge because medication adverse effects may coexist with other medical and psychiatric conditions. The differential diagnosis is particularly broad in patients with complex psychiatric comorbidities, recent alcohol withdrawal, and multiple psychotropic medication changes, where symptoms may be multifactorial and causality difficult to establish. In addition, the sympathetic nervous system plays an important role in sweat production, and α-adrenergic modulators used in psychiatric practice can alter sweating through their influence on peripheral autonomic tone [6]. The pathophysiology of medication-induced hyperhidrosis is not fully understood but is thought to involve central thermoregulatory dysfunction and increased sympathetic activity, mediated through serotonergic, adrenergic, and cholinergic systems. Given its impact on quality of life and medication adherence, recognition of possible psychotropic-induced hyperhidrosis across drug classes is essential for optimal management. This case describes a patient with persistent drenching night sweats in the setting of bipolar disorder, alcohol use disorder, multiple psychotropic medication adjustments, and a recent history of alcohol withdrawal, highlighting the diagnostic complexity of distinguishing medication-related adverse effects from alternative etiologies when no definitive cause can be established.

## Case presentation

We present the case of a 43-year-old male with a past medical history of bipolar disorder Type II, alcohol use disorder, and possible post-traumatic stress disorder (PTSD). The patient had 10 prior psychiatric inpatient hospitalizations. In October, the patient began to make complaints of drenching night sweats after alcohol cessation despite low Clinical Institute Withdrawal Assessment for Alcohol (CIWA) scores (3-7) [7].

At the onset of symptoms during his eight-day hospitalization from October 26th to November 3rd, the patient’s psychiatric medication list consisted of gabapentin 600 mg nightly and 300 mg twice daily, olanzapine 5 mg, prazosin 2 mg, and venlafaxine 150 mg. The patient continued to complain of excessive generalized night sweats “soaking through his scrubs and sheets” until his discharge, at which time his medication list consisted of gabapentin 600 mg nightly and 300 mg twice daily, hydroxyzine 25-50 mg as needed, prazosin 2 mg, and venlafaxine 150 mg.

The patient returned on November 18th after presenting to the emergency department with alcohol withdrawal and a UTI with pyelonephritis. Initial CIWA scores were above 20 on admission and improved to 10 the next day, November 19th. Following his decrease in CIWA scores, CIWA protocol was discontinued. Five days later, the patient made his first complaint of increased nighttime sweating during this hospitalization. At this time, the patient’s medication list consisted of gabapentin 300 mg, olanzapine 5 mg, prazosin 2 mg, venlafaxine 225 mg, and hydroxyzine as needed. It was decided that the patient would hold olanzapine for two days to monitor for drug side effects; however, the patient continued to complain of increased night sweats. In addition, venlafaxine was discontinued with no clinical changes. The patient’s sweating continued until December 8th, when he began to refuse any medications with the exception of hydroxyzine, which he reported decreased his symptoms. It was decided with patient consent that his current medications would be discontinued while trials of clonidine and oxcarbazepine would be attempted.

Given the persistence of night sweats despite multiple medication changes, alternative metabolic, endocrine, infectious, and malignant etiologies were considered at this time, December 9th. Physical examination revealed no lymphadenopathy, and CT imaging of the abdomen and pelvis showed no acute abnormalities or adrenal pathology. Laboratory evaluation demonstrated mild normocytic anemia and an isolated elevation in alkaline phosphatase, with otherwise unremarkable vital signs, CBC and CMP. Although glucose levels were frequently above 140 mg/dL, increased monitoring and efforts to improve glycemic control were not associated with changes in the patient's symptoms. Hormonal evaluation was notable for elevated total and free testosterone levels of 1,422 ng/dL and 331 pg/mL, respectively, although the clinical significance of these findings remained uncertain. Tuberculosis, hematologic malignancy, occult infection, and other hormonal abnormalities were also considered, but no evidence of any clear alternative etiology was identified.

A list of previous and current psychiatric medications and reported symptoms were also compiled at this time. On review of the patient's chart, he had an extensive list of psychiatric medications previously taken. This included SSRIs (fluoxetine), SNRIs (venlafaxine), benzodiazepines (lorazepam, chlordiazepoxide, diazepam), atypical antipsychotics (haloperidol, olanzapine, quetiapine), mood stabilizers (lithium), serotonin antagonist and reuptake inhibitors (SARIs; trazodone), and others (hydroxyzine, mirtazapine, melatonin, gabapentin, cyproheptadine, phenobarbital, acamprosate, diphenhydramine).

The patient’s complaints of nighttime sweating continued daily until resolving on December 18th, at which time his psychiatric medications included aripiprazole 10 mg every morning, clonidine HCl 0.1 mg, gabapentin 400 mg nightly and 300 mg twice daily, and hydroxyzine 150 mg nightly.

**Table 1:** Clinical timeline of persistent hyperhidrosis, medication changes, and diagnostic evaluation CIWA: Clinical Institute Withdrawal Assessment for Alcohol [7]

Date	Clinical course	Medication changes and interventions	Key findings
10/26–11/3	Drenching night sweats began following alcohol cessation and persisted throughout hospitalization.	At symptom onset: gabapentin 600 mg nightly and 300 mg twice daily, olanzapine 5 mg, prazosin 2 mg, and venlafaxine 150 mg.	CIWA scores remained relatively low (3–7).
11/18	Readmitted with severe alcohol withdrawal and UTI with pyelonephritis.	CIWA protocol initiated.	CIWA >20. CT abdomen/pelvis showed no acute abnormalities and normal adrenal glands.
11/19–11/21	Alcohol withdrawal symptoms improved and subsequently resolved.	CIWA protocol discontinued on 11/21.	CIWA decreased to 10 on 11/19.
11/26	First documented complaint of increased sweating during the second hospitalization.	Receiving gabapentin 300 mg, olanzapine 5 mg, prazosin 2 mg, venlafaxine 225 mg, and hydroxyzine as needed.	Sweating documented five days after discontinuation of CIWA protocol.
11/27-12/7	Night sweats persisted.	Olanzapine held for 2 days without improvement. Venlafaxine subsequently discontinued without improvement in hyperhidrosis.	Symptoms persisted despite medication adjustments.
12/8	Continued night sweats; patient began refusing all medications except hydroxyzine, which he reported decreased his symptoms.	Previous medications discontinued; trials of clonidine and oxcarbazepine subsequently attempted.	Broader medication changes undertaken because of persistent symptoms without clinical improvement
12/9	Diagnostic evaluation performed for alternative causes of persistent night sweats.	Metabolic, endocrine, infectious, and malignant etiologies considered.	Laboratory evaluation showed: mild anemia (Hgb 11.3 g/dL), elevated alkaline phosphatase (152 U/L), and elevated total and free testosterone (1,422 and 331, respectively); otherwise largely unremarkable laboratory evaluation. No lymphadenopathy.
12/18	Night sweats were no longer documented.	Receiving aripiprazole 10 mg every morning, clonidine 0.1 mg, gabapentin 400 mg nightly and 300 mg twice daily, and hydroxyzine 150 mg nightly.	No definitive causative medication or alternative etiology identified.

## Discussion

This case highlights the difficulty of attributing hyperhidrosis to a single psychotropic agent in patients with complex medication histories. Although medication-induced hyperhidrosis is well described across several psychiatric drug classes, the persistence of symptoms despite continuous medication adjustments suggests that the patient's persistent night sweats are unlikely to be attributed to one medication.

Serotonergic agents are commonly associated with hyperhidrosis through effects on central thermoregulation and sympathetic activation [1,6,8]. As such, venlafaxine was a reasonable initial target [9], however, discontinuation did not lead to meaningful change. Similarly, there were no meaningful changes in symptoms when olanzapine, another medication which has anticholinergic and autonomic side effect profiles, was discontinued. The persistence of symptoms raises the possibility of additive or sustained alterations in thermoregulatory pathways, particularly given the patient’s prolonged exposure to multiple psychotropic medications.

Application of the Naranjo Adverse Drug Reaction Probability Scale [10] demonstrated limited evidence for attributing the patient's hyperhidrosis to a single medication. Venlafaxine and olanzapine each received a score of 0, while prazosin received a score of -1, corresponding to a doubtful causal association for each medication. These assessments were limited by concurrent polypharmacy, uncertain temporal relationships between individual medication exposures and symptom onset, multiple medication changes, and the presence of alternative potential causes of hyperhidrosis.

Other potential explanations were considered. The timing of symptom onset relative to hospital admission and the patient's low CIWA scores made ongoing alcohol withdrawal less likely, and there was no clear relationship between symptoms and metabolic or infectious findings. These findings made ongoing acute alcohol withdrawal less likely to fully explain the persistence of his symptoms.

Metabolic, endocrine, infectious, and malignant etiologies were also considered. Intermittent hyperglycemia was evaluated as a potential contributor, although increased monitoring and efforts to improve glycemic control were not associated with changes in the patient's night sweats. Laboratory evaluation was notable for mild anemia, an isolated elevation in alkaline phosphatase, and markedly elevated total and free testosterone levels, although the significance of these findings in relation to the patient's symptoms remained uncertain. The remainder of the laboratory evaluation was largely unremarkable, physical examination revealed no lymphadenopathy, and available imaging showed no acute abnormalities or adrenal pathology. No clear alternative etiology was identified.

Notably, the patient reported partial symptomatic improvement with hydroxyzine, although the clinical significance of this response remains uncertain due to concurrent medication changes. In contrast, the limited benefit from clonidine suggests that reducing sympathetic outflow alone may be insufficient. Previous reports have noted varying responses to treatments such as anticholinergics, clonidine, propranolol, and calcium channel blockers, which may reflect differences in the underlying autonomic pathways contributing to symptoms [11,12]. In practice, this case shifts the focus away from identifying a single causative agent and toward managing symptoms directly. Given the impact on comfort and adherence, hyperhidrosis warrants attention even when its origin is not clearly defined.

## Conclusions

This case highlights the challenges of evaluating persistent hyperhidrosis in patients with complex psychiatric and medical histories, where multiple potential etiologies may coexist. Despite a comprehensive evaluation, no definitive cause of the patient's persistent hyperhidrosis was identified, and a medication-related contribution could neither be confirmed nor excluded. This case illustrates the importance of maintaining a broad differential diagnosis while recognizing that persistent symptoms may remain unexplained despite extensive investigation. Clinicians should continue to monitor for evolving clinical findings, reassess potential contributing factors over time, and carefully balance the therapeutic benefits of psychiatric medications against the possibility of adverse effects when managing complex presentations.
